# Proportion of Female Physicians in a Specialty and Median Annual Payments in Ontario, Canada

**DOI:** 10.1001/jamanetworkopen.2025.49815

**Published:** 2025-12-16

**Authors:** Tara Kiran, Susan E. Schultz, Rahim Moineddin, Maryam Daneshvarfard, Michelle Cohen, Lidija Latifovic, Lyn M. Sibley, Richard H. Glazier

**Affiliations:** 1 Department of Family and Community Medicine, Temerty Faculty of Medicine, University of Toronto, Toronto, Ontario, Canada; 2 Department of Family and Community Medicine, St Michael’s Hospital, Toronto, Ontario, Canada; 3 MAP Centre for Urban Health Solutions, Li Ka Shing Knowledge Institute, St Michael’s Hospital, Toronto, Ontario, Canada; 4 Institute of Health Policy, Management and Evaluation, University of Toronto, Toronto, Ontario, Canada; 5 ICES Central, Toronto, Ontario, Canada; 6 Department of Family Medicine, Queen’s University, Kingston, Ontario, Canada; 7 Dalla Lana School of Public Health, University of Toronto, Toronto, Ontario, Canada; 8 Ontario Medical Association, Toronto, Ontario, Canada; 9 Department of Family and Community Medicine, University of Toronto, Toronto, Ontario, Canada

## Abstract

**Importance:**

It is unclear why areas of medicine overrepresented by female physicians receive lower pay. One theory is devaluation—that work done by women is systematically devalued over time.

**Objective:**

To assess whether an increase in the proportion of female physicians in a specialty is associated with a decrease in the median annual payments for that specialty.

**Design, Setting, and Participants:**

In 2022, a population-based, repeated cross-sectional analysis was conducted using routinely collected data of all active physicians in Ontario, Canada, from 1992-1993 (1993) to 2019-2020 (2020). For each year, the percentage of female physicians and total annual median payments for each specialty were calculated.

**Main Outcome and Measure:**

Random-effects linear regression models were constructed to evaluate the association between the percentage of female physicians and median payments overall. Additionally, we conducted a sensitivity analysis of the subgroup of physicians with 1.0 or more full-time equivalency (FTE).

**Results:**

The physician workforce increased from 18 572 in 1993 to 31 374 in 2020 with the number of female physicians increasing from 4151 (22.3%) to 13 205 (42.1%). The mean (SD) age for all physicians in 2020 was 49.6 (12.8) years, and for female physicians in 2020 was 46.2 (11.5) years. Median annual payments increased from $183 033 to $316 570. Regression results found that an absolute 1 percentage point increase in the percentage of female physicians in a specialty over 1 year was associated with a $2183 lower increase in median payments than expected overall (95% CI, −$3932 to −$434; *P* = .02) and a $3235 lower increase for female physicians (95% CI, −$4888 to −$1583; *P* < .001), but not for male physicians (−$554; 95% CI, −$2373 to $1264).

**Conclusion and Relevance:**

This repeated cross-sectional study did not find evidence to directly support the theory of devaluation, yet the results underscore that occupational segregation, both across and within specialties, is associated with the gender pay gap. These findings should prompt medical leaders and policymakers to reconsider what activities are monetarily valued in medicine and whether these align with what patients and the public consider most valuable from a care perspective.

## Key Points

**Question:**

How is an increase in the proportion of female physicians in a specialty associated with changes in the median annual payments for that specialty?

**Findings:**

Using administrative data of all active physicians in Ontario, Canada, over nearly a 30-year period (18 572 physicians in 1993 and 31 374 in 2020), this study found that specialties with higher growth in the percentage of female physicians over time had a lower increase in total median payments overall and for female physicians but not male physicians.

**Meaning:**

These findings suggest that occupational segregation, both across and within specialties, is an important contributor to the gender pay gap.

## Teaser

This cross-sectional study evaluates whether an increase in the proportion of female physicians in a specialty is associated with a decrease in the median annual payments for that specialty.

## Introduction

Numerous studies have confirmed the existence of a gender pay gap in medicine globally. The percentage of female physicians in the workforce has risen substantially from between 10% to 20% in the 1970s to 49%, 38%, and 45% in the UK, US, and Canada, respectively, in 2022.^1,2,3,4,5,6^ Females now make up more than half of medical students in the US, Canada, and the UK.^7,8,9^ Yet, studies consistently find that, on average, male physicians earn more than female physicians even when accounting for differences in hours worked.^10^ Male physicians generally earn more than female physicians within any given specialty^11,12^ and specialties with a higher concentration of female physicians generally have lower average payments.^13,14,15^

Specifically, female physicians are overrepresented in lower paying specialties such as family medicine, pediatrics, and psychiatry while male physicians are overrepresented in higher paying specialties such as surgery and cardiology.^8,15,16,17,18,19,20,21,22^ This occupational gender segregation is sometimes referred to as pink collaring, whereby female physicians practice in areas of medicine traditionally associated with more feminine, caring traits.^23,24,25^ This phenomenon is best understood in the context of women’s historical exclusion from medicine despite taking a range of unpaid and paid roles from birth attendants to nurses. It is unclear why areas of medicine overrepresented by female physicians are lower paid, but one theory is devaluation: that work done by females is systematically devalued over time.^26,27^ This theory is supported by research showing that as occupations become more female dominated, the average pay for both men and women in that occupation tends to decline over time, even when controlling for skill and education requirements.^27^ However, little empiric research has been done to test this theory in medicine.

We used administrative data over nearly a 30-year period to understand changes in the percentage of female physicians in 36 specialty groupings and changes in median payments for those specialties and to test whether there was an association between changes in feminization of a specialty and median payments in that specialty. We hypothesized that specialties with more of an increase in female physicians over time had a lower relative increase in median payments.

## Methods

### Setting and Context

Ontario is Canada’s largest province, with 14 223 942 residents,^28^ and about 33 000 active physicians in 2021.^29^ The vast majority of physicians are paid through the Ontario Health Insurance Plan (OHIP), which provides coverage for all medically necessary physician and hospital visits for all Ontario residents. How physicians are paid changed over the study period. Before 2000, almost all physicians were paid fee-for-service. In the mid-2000s, new payment models for family physicians where introduced that incorporated some capitation and incentive payments; around the same time, alternative payment plans were introduced for some hospital-based specialties, such as emergency medicine and oncology. Today, about 46% of family physicians’ payments are through fee-for-service while 81% of payments to other specialists are fee-for-service.^30^ Reporting of this repeated cross-sectional study follows the Strengthening the Reporting of Observational Studies in Epidemiology (STROBE) reporting guideline. The use of the data in this project is authorized under section 45 of Ontario’s Personal Health Information Protection Act and does not require review by a research ethics board. Informed consent is also not required.

### Study Design and Population

Using administrative data, we conducted a repeated cross-sectional analysis of all active Ontario physicians from fiscal year 1992-1993 (1993) to fiscal year 2019-2020 (2020) to investigate changes in the proportion of female physicians in each specialty, changes in median payments, and the association between these. We also assessed changes in the size of the gender pay gap over time. To identify active physicians, we used a derived database created at ICES using specialty and practice location information from the Ontario Physician Human Resource Data Centre. We included physicians with a valid Ontario postal code who had payments in at least 1 of the payment databases held at ICES for the given year. We excluded physicians who were out of province, clinical fellows, those with missing information on gender and date of birth, and physicians whose total payments for the year were negative.

Datasets were linked using unique encoded identifiers and analyzed at ICES. A full list of datasets and variables is summarized in eTable 1 in Supplement 1. ICES is an independent, nonprofit research institute whose legal status under Ontario’s health information privacy law allows it to collect and analyze health care and demographic data, without consent, for health system evaluation and improvement.

#### Physician Demographics, Practice Characteristics, and Full-Time Equivalency

Physician demographic information was obtained from the OHIP Corporate Provider Database. Data on physician sex originates from the College of Physicians and Surgeons of Ontario and is based on physician self-report of whether they identify as male or female (an option for other was added in 2021). The terms male and female are used throughout for consistency with the data available. Physicians’ full-time equivalencies (FTEs) were calculated using an algorithm from the Canadian Institute for Health Information,^31^ which determines a physician’s FTE based on their total payments relative to the upper and lower payment benchmarks for their specialty. The 40% to 60% range of the distribution represents a full-time practice (FTE = 1.0), while payments exceeding 60% indicate a practice with an FTE greater than 1.0. No data were available across specialties on hours worked. Each unique combination of physician, patient, and day was counted as a visit. Any day where physicians saw a minimum of 1 patient was counted as a day worked. We used the rurality index of Ontario score to classify the rurality of a physician’s practice with a score of 0, 1 to 9, 10 to 39, and 40 denoting big cities, small cities, small towns, and rural communities, respectively.^32^

#### Specialty Groupings

Physicians were classified into specialty groups according to their derived specialty in the ICES Physician Database. Specialties with fewer than 20 physicians were merged with larger, related specialties. All pediatric subspecialties, except for pediatric surgery, were grouped together. Clinical fellows and out-of-province physicians were excluded from the FTE estimates (eTable 2 in Supplement 1).

#### Median Payments by Specialty

We calculated physicians’ total payments from all Ministry of Health sources using data from 3 sources: the OHIP database of fee-for-service payments; the Architected Payments database of premiums, bonuses, and other incentives associated with OHIP; and the Generic Alternate Payment Program database^33^ of alternative payment plan payments to groups, alternative funding arrangement payments to physicians working in the emergency department, and various miscellaneous payments. When estimating payment totals, we excluded payments that could not be allocated to individual physicians, claims that included dummy physician numbers or were duplicate, and technical fees. Total payments to individual physicians within a specialty were used to compute the median total payment for that specialty each year from 1993 to 2020. Payment per visit was estimated by dividing the total payments from all sources by the total number of visits for each physician and year.

### Statistical Analysis

To estimate the size of the gender pay gap within and across specialties, we calculated the median total payments per physician, stratified by gender, and the ratio of payments to male vs female physicians in each grouped specialty from 1993 to 2020. To understand trends over time, we plotted changes in the percentage of females and median payments over time separately for each specialty. We constructed random-effects linear regression models (incorporating both random intercept and random slope) with autoregressive (AR[1]) model for residuals to evaluate the association between the percentage of female physicians and median payments. In these models, specialty served as the unit of analysis with repeated measures across different years. We included year and the percentage of female physicians as variables, while median payments was the outcome variable. To account for variability among specialties, we applied random effects, and to address serial correlation within specialties, we used an AR(1) model. The analysis was conducted using the MIXED procedure in SAS to model and estimate the parameters. We stratified the regression analysis to understand how changes in the percentage of female physicians were associated with median payments for male and female physicians separately. We performed a sensitivity analysis restricted to physicians with an FTE value greater than or equal to 1, thereby excluding physicians who worked very part-time. *P* values were obtained from regression models. All statistical tests were 2-sided, and statistical significance was defined as α = .05. All analyses were performed in SAS version 9.4 (SAS Institute); figures were generated in R version 4.0.5 (R Project for Statistical Computing) and Excel version 16.0.5526.1002 (Microsoft).

## Results

### Demographic and Practice Characteristics of Female and Male Physicians

The physician workforce increased from 18 572 in 1993 to 31 374 (a 69% increase) in 2020 with the number of female physicians increasing from 4151 (22.4%) to 13 205 (42.1%; a 219% increase) (Table 1). Through the study period, female physicians were younger, graduating 6 to 7 years later (mean [SD] in 1993: male, 1970 [12.1]; female, 1977 [10.0]; in 2020: male, 1993 [13.9]; female, 1999 [11.9]). The mean (SD) age for all physicians in 2020 was 49.6 (12.8) years, and for female physicians in 2020 was 46.2 (11.5) years. Compared with male physicians, a higher proportion of female physicians specialized in family medicine (eg, 6714 [50.8%] vs 7018 [38.6%] in 2020) and fewer worked an FTE or more (eg, 6302 [47.7%] vs 11 368 [62.5%] in 2020). The mean (SD) number of days worked by all physicians decreased from 239.8 (80.6) in 1993 to 204.4 (75.3; a decrease of 15%) in 2020. Throughout the study period, female physicians worked fewer days (mean [SD], 191.3 [73.6] vs 213.9 [75.1] days in 2020), had fewer patient visits per year (mean [SD], 3161.7 [3165.8] vs 4578.7 [5094.8] visits in 2020), and had fewer patient visits per day (mean [SD], 15.1 [10.8] vs 19.5 [16.5] visits in 2020) compared with male physicians. Compared with male physicians, female physicians also had lower total payments (mean [SD], $289 960 [$208 112] vs $420 579 [$315 351] in 2020), lower payments per day worked (mean [SD], $1494 [$1059] vs $1912 [$1244] in 2020) and lower payment per patient visit (mean [SD], $134 [$150] vs $139 [$134] in 2020). Trends were similar for the subgroup of physicians with an FTE of 1.0 or more with female physicians having lower payments per day worked (mean [SD], $1905 [$1233] vs $2324 [$1298] in 2020) and lower payment per patient visit (mean [SD], $139 [$159] vs $142 [$128] in 2020) (eTable 3 in Supplement 1).

**Table 1.** Characteristics of all Physicians in 1992-1993, 2005-2006, and 2019-2020, Stratified by Sex

Characteristic	Physicians, No. (%)								
	1992-1993			2005-2006			2019-2020		
	Female (n = 4151)	Male (n = 14 421)	Total (n = 18 572)	Female (n = 6574)	Male (n = 14 927)	Total (n = 21 501)	Female (n = 13 205)	Male (n = 18 169)	Total (n = 31 374)
Age, mean (SD), y	40.6 (9.8)	47.3 (12.1)	45.8 (12.0)	45.3 (10.0)	51.6 (11.9)	49.7 (11.7)	46.2 (11.5)	52.0 (13.2)	49.6 (12.8)
Age group									
<40 y	2291 (55.2)	4540 (31.5)	6831 (36.8)	2105 (32.0)	2716 (18.2)	4821 (22.4)	4676 (35.4)	3846 (21.2)	8522 (27.2)
40-54 y	1446 (34.8)	5797 (40.2)	7243 (39.0)	3338 (50.8)	6305 (42.2)	9643 (44.8)	5248 (39.7)	6571 (36.2)	11 819 (37.7)
55-64 y	270 (6.5)	2395 (16.6)	2665 (14.3)	821 (12.5)	3356 (22.5)	4177 (19.4)	2117 (16.0)	3654 (20.1)	5771 (18.4)
≥65 y	124 (3.0)	1474 (10.2)	1598 (8.6)	264 (4.0)	2319 (15.5)	2583 (12.0)	961 (7.3)	3667 (20.2)	4628 (14.8)
Missing	20 (0.5)	215 (1.5)	235 (1.3)	46 (0.7)	231 (1.5)	277 (1.3)	203 (1.5)	431 (2.4)	634 (2.0)
Graduation year, mean (SD)	1977 (10.0)	1970 (12.1)	1972 (12.0)	1985 (10.4)	1979 (12.2)	1981 (12.1)	1999 (11.9)	1993 (13.9)	1996 (13.4)
No. of years in practice, mean (SD)	14.8 (10.0)	21.6 (12.1)	20.0 (12.0)	19.1 (10.4)	25.8 (12.2)	23.7 (12.1)	19.5 (11.9)	25.7 (13.9)	23.1 (13.4)
Rurality									
Major urban (0)	2867 (69.1)	8739 (60.6)	11 606 (62.5)	4342 (66.0)	8867 (59.4)	13 209 (61.4)	8157 (61.8)	10 493 (57.8)	18 650 (59.4)
Urban (1-9)	726 (17.5)	2798 (19.4)	3524 (19.0)	1274 (19.4)	3332 (22.3)	4606 (21.4)	3137 (23.8)	4592 (25.3)	7729 (24.6)
Suburban (10-39)	381 (9.2)	2037 (14.1)	2418 (13.0)	669 (10.2)	1995 (13.4)	2664 (12.4)	1365 (10.3)	2289 (12.6)	3654 (11.6)
Rural (≥40)	149 (3.6)	753 (5.2)	902 (4.9)	272 (4.1)	694 (4.6)	966 (4.5)	516 (3.9)	759 (4.2)	1275 (4.1)
Missing	28 (0.7)	94 (0.7)	122 (0.7)	17 (0.3)	39 (0.3)	56 (0.3)	30 (0.2)	36 (0.2)	66 (0.2)
FTE (grouped)									
<0.5	1115 (26.9)	2524 (17.5)	3639 (19.6)	1411 (21.5)	2150 (14.4)	3561 (16.6)	2738 (20.7)	2696 (14.8)	5434 (17.3)
0.5 to <1.0	1486 (35.8)	2955 (20.5)	4441 (23.9)	2382 (36.2)	3170 (21.2)	5552 (25.8)	4165 (31.5)	4105 (22.6)	8270 (26.4)
1.0	800 (19.3)	2717 (18.8)	3517 (18.9)	1362 (20.7)	2773 (18.6)	4135 (19.2)	2771 (21.0)	3096 (17.0)	5867 (18.7)
>1.0 to 1.2	374 (9.0)	2461 (17.1)	2835 (15.3)	704 (10.7)	2551 (17.1)	3255 (15.1)	1577 (11.9)	2597 (14.3)	4174 (13.3)
>1.2	376 (9.1)	3764 (26.1)	4140 (22.3)	715 (10.9)	4283 (28.7)	4998 (23.2)	1954 (14.8)	5675 (31.2)	7629 (24.3)
Specialty									
Family medicine	2527 (60.9)	6480 (48.6)	9275 (49.9)	3467 (52.7)	6210 (41.6)	9677 (45.0)	6714 (50.8)	7018 (38.6)	13 732 (43.8)
All other specialties	1624 (39.1)	7673 (53.2)	9297 (50.1)	3107 (47.3)	8717 (58.4)	11 824 (55.0)	6491 (49.2)	11 151 (61.4)	17 642 (56.2)
Days worked per y^a^									
Mean (SD)	211.6 (76.7)	247.9 (79.9)	239.8 (80.6)	195.8 (71.3)	226.6 (73.5)	217.2 (74.2)	191.3 (73.6)	213.9 (75.1)	204.4 (75.3)
Median (IQR)	227 (168-267)	263 (215-305)	255 (203-298)	209 (149-247)	239 (190-277)	231 (175-268)	200 (140-246)	225 (169-268)	215 (155-258)
Patient visits per y									
Mean (SD)	3787.6 (3164.8)	5453.8 (4410.9)	5081.4 (4222.3)	3720.5 (3253.9)	5425.2 (4777.1)	4904.0 (4438.1)	3161.7 (3165.8)	4578.7 (5094.8)	3982.3 (4442.9)
Median (IQR)	3121 (1284-5448)	4660 (1991-7821)	4257 (1776-7264)	2945 (1334-5256)	4288 (2004-7655)	3815 (1747-6844)	2433 (1224-4057)	3370 (1717-5650)	2925 (1477-4949)
Patient visits per d									
Mean (SD)	16.6 (11.2)	20.5 (14.3)	19.6 (13.7)	17.4 (11.5)	22.1 (15.9)	20.7 (14.8)	15.1 (10.8)	19.5 (16.5)	17.6 (14.6)
Median (IQR)	15 (8-22)	18 (10-28)	18 (9-26)	16 (9-23)	19 (11-30)	18 (10-28)	13 (9-18)	16 (10-23)	14 (9-21)
Total payments from all sources									
Mean (SD)	143 308 (103 555)	216 998 (143 637)	200 527 (139 136)	200 650 (133 822)	297 698 (189 645)	268 025 (180 118)	289 960 (208 112)	420 579 (315 351)	365 603 (282 800)
Median (IQR)	129 219 (71 132-188 708)	202 136 (120 180-290 474)	183 033 (102 153-269 519)	176 378 (107 488-266 138)	275 072 (161 977-394 971)	241 462 (135 439-360 040)	259 479 (151 331-387 604)	374 639 (208 575-552 794)	316 570 (179 729-485 908)
Payment per day worked^a^									
Mean (SD)	643 (367)	837 (476)	794 (461)	1022 (678)	1294 (768)	1211 (752)	1494 (1059)	1912 (1244)	1736 (1188)
Median (IQR)	586 (424-776)	773 (548-1043)	723 (508-991)	877 (636-1250)	1166 (821-1600)	1074 (743-1504)	1343 (947-1842)	1708 (1162-2371)	1538 (1053-2148)
Payment per patient visit									
Mean (SD)	52 (43)	55 (47)	55 (46)	88 (142)	89 (107)	89 (119)	134 (150)	139 (134)	137 (141)
Median (IQR)	33 (28-64)	38 (27-69)	37 (27-68)	52 (36-104)	59 (35-108)	57 (35-107)	100 (65-155)	104 (66-162)	103 (65-159)

Abbreviation: FTE, full-time equivalency.

^a^ A day worked was defined as any day on which a physician visited a minimum of 1 patient.

### Changes in the Size of the Gender Pay Gap Within Specialties Using Median Annual Payments

**Figure 1.**
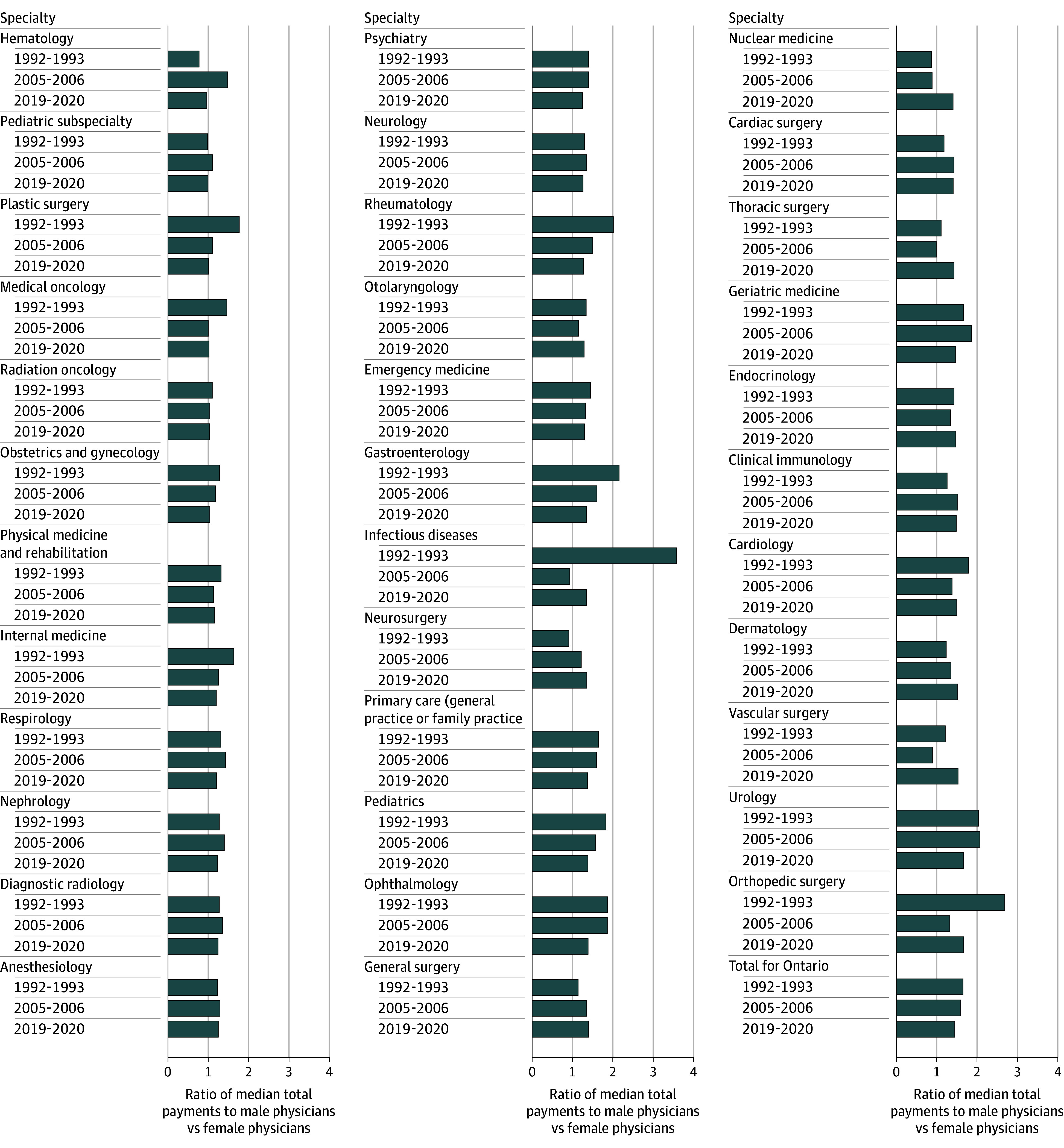
Number of Physicians and the Ratio of Median Total Payments to Male Physicians vs Female Physicians in 1992-1993, 2005-2006, and 2019-2020, Stratified by Specialty

Median total annual payments for female physicians were lower than male physicians for almost every specialty in every time period (the ratio of median total payments to male vs female physicians >1), although the magnitude of the difference decreased overall with time (Figure 1). In 2020, male physicians were paid $1.45 for each dollar paid to female physicians, compared with $1.65 in 1993. Hematology was the only specialty where female physicians were not paid less. The largest gap was in orthopedic surgery and urology, where male physicians were paid $1.67 for each dollar paid to female physicians. Among physicians working an FTE of 1.0 or more, the overall size of the gender pay gap did not change substantially over time. In 2020, male physicians were paid $1.30 for each dollar paid to female physicians, compared with $1.27 in 1993. For more than half of the specialties, the size of the gap was greater in 2020 compared with 1993 (eFigure 1 in Supplement 1).

### Changes in the Percentage of Female Physicians and Median Total Payments by Specialty

The percentage of female physicians and the median payments increased in all specialties over the study period overall (eFigure 2 in Supplement 1) and in the subgroup of physicians with an FTE of 1.0 or more (eFigure 3 in Supplement 1). In 2020, pediatrics had the highest percentage of female physicians (1426 physicians [61.2%]) and urology had the lowest (318 physicians [7.9%]) while diagnostic radiology had the highest median payments ($622 833) and psychiatry had the lowest ($186 547).

**Figure 2.**
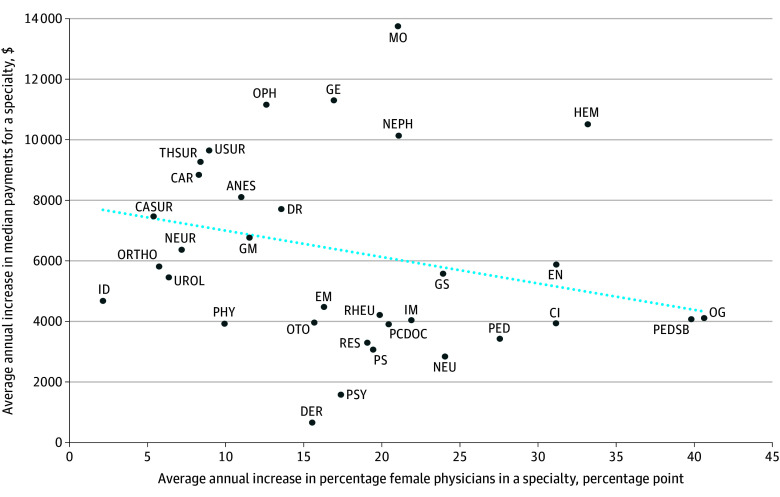
Association Between Mean Annual Changes in Median Payments and Percentage of Female Physicians by Physician Specialty, 1992-1993 to 2019-2020, for All Physicians ANES indicates anesthesiology; CAR, cardiology; CASUR, cardiac surgery; CI, clinical immunology; DER, dermatology; DR, diagnostic radiology; EM, emergency medicine; EN, endocrinology; GE, gastroenterology; GM, geriatric medicine; GS, general surgery; HEM, hematology; ID, infectious diseases; IM, internal medicine; MO, medical oncology; NEPH, nephrology; NEU, neurology; NEUR, neurosurgery; OG, obstetrics and gynecology; OPH, ophthalmology; ORTHO, orthopedics; OTO, otolaryngology; PCDOC, primary care physician; PED, pediatrics; PEDSB, pediatric subspecialty; PHY, physical medicine and rehabilitation; PS, plastic surgery; PSY, psychiatry; RES, respirology; RHEU, rheumatology; THSUR, thoracic surgery; UROL, urology; USUR, vascular surgery.

Figure 2 depicts every specialty and the corresponding change in the percentage of female physicians and change in median payments during the study time period, showing an overall negative association. Regression analysis confirmed a negative association between the change in the percentage of female physicians and change in median payments in a given specialty (Table 2). Specifically, on average, each 1 percentage point (PP) increase in the percentage of female physicians in a specialty was associated with a $2183 lower increase in median payments than expected in a 1-year time period (95% CI, $434 to $3932; *P* = .02). Results were similar for physicians with an FTE of 1.0 or greater, with a 1 PP increase in the percentage of female physicians associated with a $2107 lower increase in median payments over one year (95% CI, $335 to $3879; *P* = .02).

**Table 2.** Regression Analysis Results for the Association Between the Change in the Percentage of Female Physicians and the Change in Median Payments Over Time

Physician type	Estimate (95% CI)^a^	*P* value
All		
Overall	−$2183 (−$3932 to −$434)	.02
FTE ≥1.0	−$2107 (−$3879 to −$335)	.02
Female		
Overall	−$3235 (−$4888 to −$1583)	<.001
FTE ≥1.0	−$3182 (−$4874 to −$1489)	.001
Male		
Overall	−$554 (−$2373 to $1264)	.54
FTE ≥1.0	−$454 (−$2283 to $1375)	.62

Abbreviation: FTE, full-time equivalent.

^a^ Estimates indicate the change in median payments in dollars for a 1 percentage point increase in the percentage of female physicians in a 1-year time period in a specialty.

Stratified regression analyses demonstrated a different effect of feminization of a specialty on the payments of the female and male physician workforce. For female physicians, a 1 PP increase in the percentage of female physicians in the specialty was associated with a $3235 lower increase in median payments than expected over 1 year (95% CI, $1583 to $4888; *P* < .001). However, for male physicians, there was no significant association between changes in the percentage of female physicians in a specialty and median payments (−$554; 95% CI, −$2373 to $1264; *P* = .54). These findings were also consistent for physicians with FTE of 1.0 or more.

## Discussion

Between 1993 to 2020, the physician workforce in Ontario, Canada, grew about 70% with the percentage of female physicians increasing from 22% to 42%. Median physician payments also increased about 70%, not accounting for inflation, during the same time period. The overall size of the gender pay gap, as measured by median physician payments, decreased from $1.65 in 1993 to $1.45 in 2020 in payments for male physicians for every $1 earned by female physicians. The gap was present in nearly all specialties with surgical subspecialties having some of the largest gaps. We found that specialties with higher growth in the percentage of female physicians had less of an increase in total median payments during the study period. An absolute 1 PP increase in the percentage of female physicians in a specialty over 1 year was associated with a $2183 lower increase in median payments than expected. Extrapolated over 10 years, where there was an absolute 1 PP increase in the percentage of female physicians per year, this would correspond to median payments that are $21 830 less than expected for the specialty.

Importantly, we found that the increase in the percentage of female physicians in a specialty was associated with lower increases in payments overall and in payments to female physicians but not payments to male physicians. Unlike the study by Pelley and colleagues,^26^ our findings do not directly support the hypothesis of devaluation, which would result in decreases in payments to both female and male physicians. Instead, it adds to the evidence demonstrating both vertical and horizontal segregation in medicine and the complex intersection between these.

It is unclear why we found different associations for male and female physicians. Male physicians may have been protected from devaluation due to relative seniority.^34,35^ Other factors influencing differences in pay within a specialty—or horizontal segregation—include referral bias,^36,37^ time spent per patient,^38,39,40,41^ patient expectations,^42,43^ access to resources,^51^ and the monetary value assigned to different types of work^44,45,46^ (for example, remuneration for volume instead of time spent per patient). Importantly, our study could not determine causation. For example, when looking at changes in gender composition and pay between specialties—or vertical segregation—it is unclear whether increasing female representation led to declining pay through specific mechanisms (eg, fee codes) or whether lower-paying specialties (and lower-paying work within those specialties) were less attractive to men. Unpacking these factors and how they influence both horizontal and vertical occupational segregation are important avenues for future research.

Some of the gender pay gap noted in our study may be explained by female physicians working fewer days per year, seeing fewer patients per day, and being younger than male colleagues. However, the payment per patient visit was lower for female than male physicians suggesting that part-time work does not fully explain the gap. Moreover, prior research consistently shows that gender pay gaps in medicine persist even after adjustment for factors such as age and experience.^13,17^ Research that builds on ours using payment per visit instead of annual median payments may provide further insights. Our findings were consistent for the subpopulation of physicians considered to work 1 FTE; however, FTE was determined by payments as we did not have access to hours worked in our dataset. Finally, although differences in payment increases by specialty may seem small relative to overall earnings, the cumulative impact of lower increases over time is substantial.

### Limitations

Our study has limitations. We did not have data on hours worked but used a validated algorithm based on payments to estimate FTE. There were some missing payment data between 2000 and 2004, when specific specialties were switching to an alternative payment plan. We also did not have data on payments made by patients out-of-pocket or via private insurance; these would be a very small percentage of physician income overall but influence estimates for specialties such as dermatology where physicians commonly charge privately for services not covered under provincial health insurance (eg, cosmetic procedures).^47^ Our analysis includes all specialties over a 27-year time period, but more research is needed for each specific specialty to understand contributors to the gender pay gap, including potential differences in practice patterns, patients served, and compensation models. We conducted a regression analysis using aggregated data by specialty and were not able to adjust for factors such as years of experience and rurality which are important avenues for future research. The regression was also limited by the number of specialties (n = 36) and this sample size, although complete, would have influenced confidence intervals. We did not adjust for inflation, which would affect the magnitude but not direction of trends. Most importantly, our analysis was limited to differences between female and male physicians; we did not have data on other genders, race, ethnicity, disability, or other factors that intersect to widen payment disparities.

## Conclusions

In this repeated cross-sectional study of physician payments in Ontario, Canada, in all specialty groupings over almost 3 decades, we found that an increase in the percentage of female physicians in a specialty was associated with a decrease in median payments overall and for female physicians but not male physicians. Our results do not directly support the hypothesis of devaluation as a contributor to the gender pay gap in medicine; instead, they underscore the complex interplay of vertical and horizontal occupational gender segregation in medicine. Our findings should prompt medical leaders and policymakers to reconsider what activities are monetarily valued in medicine and whether these align with what patients and the public consider most valuable from a care perspective. Research suggests women, on average, spend more time with patients,^39,40^ see more medically and socially complex patients,^48^ provide more guideline-based care^49^ and have patients with better outcomes.^40,50^ However, current payment models that emphasize volume may inadequately capture the full scope and value of physicians’ work. More research is needed to understand how these practice differences and our implicit biases are contributing to the gender pay gap.

## Notes

Supplement 1. eTable 1. ICES Data Sources Used in the Study eTable 2. ICES Grouping of Specialties eTable 3. Characteristics of All Full-Time Physicians in 1992-1993, 2005-2006, and 2019-2020, Stratified by Sex eFigure 1. Ratio of Median Total Payments to Male Physicians vs Female Physicians in Full-Time Physicians in 1992-1993, 2005-2006, and 2019-2020, Stratified by Specialty eFigure 2. Changes in the Percentage of Female Physicians and Median Total Payment in All Physicians in All Years From 1992-1993 to 2019-2020, by SpecialtyeReferences eFigure 3. Changes in the Percentage of Female Physicians and Median Total Payment in Full-Time Physicians From 1992-1993 to 2019-2020, by Specialty

Supplement 2. Data Sharing Statement
