# Supplementary material for: Proportion of Female Physicians in a Specialty and Median Annual Payments in Ontario, Canada
Source: JAMA Netw Open. 2025 Dec 16;8(12):e2549815. doi: 10.1001/jamanetworkopen.2025.49815 (PMC12709378; doi:10.1001/jamanetworkopen.2025.49815)
Supplement: Supplement 1. — eTable 1. ICES Data Sources Used in the Study eTable 2. ICES Grouping of Specialties eTable 3. Characteristics of All Full-Time Physicians in 1992-1993, 2005-2006, and 2019-2020, Stratified by Sex eFigure 1. Ratio of Median Total Payments to Male Physicians vs Female Physicians in Full-Time Physicians in 1992-1993, 2005-2006, and 2019-2020, Stratified by Specialty eFigure 2. Changes in the Percentage of Female Physicians and Median Total Payment in All Physicians in All Years From 1992-1993 to 2019-2020, by SpecialtyeReferences eFigure 3. Changes in the Percentage of Female Physicians and Median Total Payment in Full-Time Physicians From 1992-1993 to 2019-2020, by Specialty [file jamanetwopen-e2549815-s001.pdf]

Supplemental Online Content

Kiran T, Schultz S, Moineddin R, et al. Proportion of female physicians in a specialty and median annual payments in Ontario, Canada. *JAMA Netw Open*. 2025;8(12):e2549815. doi:10.1001/jamanetworkopen.2025.49815

eTable 1. ICES Data Sources Used in the Study

eTable 2. ICES Grouping of Specialties

eTable 3. Characteristics of All Full-Time Physicians in 1992-1993, 2005-2006, and 2019-2020, Stratified by Sex

eFigure 1. Ratio of Median Total Payments to Male Physicians vs Female Physicians in Full-Time Physicians in 1992-1993, 2005-2006, and 2019-2020, Stratified by Specialty

eFigure 2. Changes in the Percentage of Female Physicians and Median Total Payment in All Physicians in All Years From 1992-1993 to 2019-2020, by SpecialtyeReferences

eFigure 3. Changes in the Percentage of Female Physicians and Median Total Payment in Full-Time Physicians From 1992-1993 to 2019-2020, by Specialty

This supplemental material has been provided by the authors to give readers additional information about their work.

© 2025 Kiran T et al. *JAMA Netw Open*.

eTable 1. ICES Data Sources Used in the Study
https://datadictionary.ices.on.ca/Applications/DataDictionary/Default.aspx

Database name	Description & Variables
ICES Physician Database (IPDB)	This care provider database includes information from Ontario physicians, providing details on physician characteristics and metrics of physician activity. We used the ICES Physician Database to identify active physicians.
OHIP Corporate Provider Database (CPDB)	This care provider database includes information about providers who are eligible for OHIP payments, providing details on provider demographics, training, and practice locations. We used the OHIP Corporate Provider Database to ascertain physicians' demographic information.
Ontario Health Insurance Plan Claims Database (OHIP)	This health services database includes information about billing claims submitted by Ontario physicians. Physicians operating under non-fee-for-service models submit shadow billings to OHIP, which are recorded as billing claims with a payment value of $0. We used the Ontario Health Insurance Plan Claims Database to determine practice information and physician's payments.
Architected Payments Database	This database includes premiums, bonuses, and other incentives associated with OHIP. We used the Architected Payments Database to determine physician's payments.
Generic Alternate Payment Program (GAPP) Database of payments made through Alternate Payment Plans (APPs)	This database includes payments from a number of sources, but are primarily non-FFS payments to groups, alternate funding arrangement payments to physicians working in the Emergency Department and various miscellaneous payments, discounts and adjustments. We used the Generic Alternate Payment Program Database to determine physician's payments.

© 2025 Kiran T et al. *JAMA Netw Open*.

eTable 2. ICES Grouping of Specialties (https://www.ices.on.ca/).

MAIN SPECIALTY	SPECIALTY GROUPING
ANATOMICAL PATHOLOGY<br>FORENSIC PATHOLOGY	ANATOMICAL PATHOLOGY
ANESTHESIOLOGY<br>PAIN MEDICINE	ANESTHESIOLOGY
CARDIAC SURGERY	CARDIAC SURGERY
CARDIOLOGY	CARDIOLOGY
CLINICAL IMMUNOLOGY	CLINICAL IMMUNOLOGY
CRITICAL CARE MEDICINE	CRITICAL CARE MEDICINE
DERMATOLOGY	DERMATOLOGY
DIAGNOSTIC RADIOLOGY	DIAGNOSTIC RADIOLOGY
EMERGENCY MEDICINE<br>F.P./EMERGENCY MEDICINE	EMERGENCY MEDICINE
ENDOCRINOLOGY	ENDOCRINOLOGY
GASTROENTEROLOGY	GASTROENTEROLOGY
GENERAL PATHOLOGY<br>HEMATOLOGICAL PATHOLOGY<br>NEUROPATHOLOGY	GENERAL PATHOLOGY
COLORECTAL SURGERY<br>GENERAL SURGERY<br>PEDIATRIC SURGERY<br>SURGICAL ONCOLOGY	GENERAL SURGERY
GERIATRIC MEDICINE	GERIATRIC MEDICINE
HEMATOLOGY	HEMATOLOGY
INFECTIOUS DISEASES	INFECTIOUS DISEASES
CLINICAL PHARMACOLOGY<br>INTERNAL MEDICINE<br>PALLIATIVE MEDICINE	INTERNAL MEDICINE
LAB MEDICINE<br>MEDICAL BIOCHEMISTRY<br>MEDICAL MICROBIOLOGY	LAB MEDICINE
MEDICAL ONCOLOGY	MEDICAL ONCOLOGY
NEPHROLOGY	NEPHROLOGY
NEUROLOGY	NEUROLOGY
NEUROSURGERY	NEUROSURGERY
NUCLEAR MEDICINE	NUCLEAR MEDICINE
GYNECOLOGIC ONCOLOGY<br>MATERNAL FETAL MEDICINE<br>OBSTETRICS AND GYNECOLOGY<br>REPRODUCTIVE ENDOCRINOLOGY	OBSTETRICS AND GYNECOLOGY

© 2025 Kiran T et al. *JAMA Netw Open*.

OCCUPATIONAL MEDICINE	OCCUPATIONAL MEDICINE
OPHTHALMOLOGY	OPHTHALMOLOGY
ORTHOPEDIC SURGERY	ORTHOPEDIC SURGERY
OTOLARYNGOLOG	OTOLARYNGOLOG
COMMUNITY MED./PUBLIC HEALTH<br>GP/FP	PC DOC
PEDIATRIC CARDIOLOGY<br>PEDIATRIC CLINICAL IMMUNOLOGY<br>PEDIATRIC CRITICAL CARE<br>PEDIATRIC EMERGENCY MEDICINE<br>PEDIATRIC ENDOCRINOLOGY<br>PEDIATRIC GASTROENTEROLOGY<br>PEDIATRIC HEMATOLOGY<br>PEDIATRIC INFECTIOUS DISEASES<br>PEDIATRIC NEPHROLOGY<br>PEDIATRIC NEUROLOGY<br>PEDIATRIC RADIOLOGY<br>PEDIATRIC RESPIROLOGY<br>PEDIATRIC RHEUMATOLOGY	PEDIATRIC SUBSPEC
ADOLESCENT MEDICINE<br>DEVELOPMENTAL PEDIATRICS<br>MEDICAL GENETICS<br>NEONATAL/PERINATAL MEDICINE<br>PEDIATRICS	PEDIATRICS
PHYSICAL MEDICINE AND REHAB	PHYSICAL MEDICINE AND REHAB
PLASTIC SURGERY	PLASTIC SURGERY
CHILD & ADOLESCENT PSYCHIATRY<br>FORENSIC PSYCHIATRY<br>GERIATRIC PSYCHIATRY<br>PSYCHIATRY	PSYCHIATRY
RADIATION ONCOLOGY	RADIATION ONCOLOGY
RESPIROLOGY	RESPIROLOGY
RHEUMATOLOGY	RHEUMATOLOGY
THORACIC SURGERY	THORACIC SURGERY
UROLOGY	UROLOGY
VASCULAR SURGERY	VASCULAR SURGERY

eTable 3. Characteristics of All Full-Time Physicians in 1992-1993, 2005-2006, and 2019-2020, Stratified by Sex

	1992/93			2005/2006			2019/2020		
	**Female**	**Male**	**Total**	**Female**	**Male**	**Total**	**Female**	**Male**	**Total**
**No. physicians**	1,550	8,942	10,492	2,781	9,607	12,388	6,302	11,368	17,670
**Age, Mean ± SD**	41.1 ± 8.4	45.9 ± 10.1	45.1 ± 10.0	46.1 ± 9.1	49.8 ± 10.2	49.0 ± 10.1	46.9 ± 10.3	51.0 ± 11.7	49.6 ± 11.4
**Age group, N (%)**									
<40	767 (49.5%)	2,748 (30.7%)	3,515 (33.5%)	728 (26.2%)	1,784 (18.6%)	2,512 (20.3%)	1,801 (28.6%)	2,199 (19.3%)	4,000 (22.6%)
40-54	669 (43.2%)	4,267 (47.7%)	4,936 (47.0%)	1,560 (56.1%)	4,611 (48.0%)	6,171 (49.8%)	2,956 (46.9%)	4,733 (41.6%)	7,689 (43.5%)
55-64	90 (5.8%)	1,443 (16.1%)	1,533 (14.6%)	406 (14.6%)	2,312 (24.1%)	2,718 (21.9%)	1,106 (17.5%)	2,541 (22.4%)	3,647 (20.6%)
65+	22 (1.4%)	380 (4.2%)	402 (3.8%)	70 (2.5%)	766 (8.0%)	836 (6.7%)	347 (5.5%)	1,635 (14.4%)	1,982 (11.2%)
Missing	<=5 (0.1%)	104 (1.2%)	106 (1.0%)	17 (0.6%)	134 (1.4%)	151 (1.2%)	92 (1.5%)	260 (2.3%)	352 (2.0%)
**Graduation year, Mean ± SD**	1976 ± 8.7	1971 ± 10.2	1972 ± 10.1	1984 ± 9.6	1980 ± 10.5	1981 ± 10.5	1998 ± 10.8	1994 ± 12.3	1995 ± 12.0
**No. years in practice, Mean ± SD**	15.4 ± 8.7	20.2 ± 10.2	19.5 ± 10.1	20.1 ± 9.6	24.0 ± 10.5	23.2 ± 10.5	20.4 ± 10.8	24.7 ± 12.3	23.2 ± 12.0
**Rurality (RIO), N (%)**									
Major Urban (0)	987 (63.7%)	4,975 (55.6%)	5,962 (56.8%)	1,763 (63.4%)	5,401 (56.2%)	7,164 (57.8%)	3,681 (58.4%)	6,259 (55.1%)	9,940 (56.3%)
Urban (1-9)	355 (22.9%)	2,021 (22.6%)	2,376 (22.6%)	611 (22.0%)	2,431 (25.3%)	3,042 (24.6%)	1,665 (26.4%)	3,208 (28.2%)	4,873 (27.6%)
Suburban (10-39)	161 (10.4%)	1,444 (16.1%)	1,605 (15.3%)	314 (11.3%)	1,383 (14.4%)	1,697 (13.7%)	730 (11.6%)	1,511 (13.3%)	2,241 (12.7%)
Rural (40+)	45 (2.9%)	481 (5.4%)	526 (5.0%)	91 (3.3%)	380 (4.0%)	471 (3.8%)	217 (3.4%)	382 (3.4%)	599 (3.4%)
Missing	<=5 (0.1%)	21 (0.2%)	23 (0.2%)	<=5 (0.1%)	12 (0.1%)	14 (0.1%)	9 (0.1%)	8 (0.1%)	17 (0.1%)
**FTE (grouped), N (%)**									
FTE = 1.0	800 (19.3%)	2,717 (18.8%)	3,517 (18.9%)	1,362 (49.0%)	2,773 (28.9%)	4,135 (33.4%)	2,771 (44.0%)	3,096 (27.2%)	5,867 (33.2%)
FTE >1.0-1.2	374 (9.0%)	2,461 (17.1%)	2,835 (15.3%)	704 (25.3%)	2,551 (26.6%)	3,255 (26.3%)	1,577 (25.0%)	2,597 (22.8%)	4,174 (23.6%)
FTE >1.2	376 (9.1%)	3,764 (26.1%)	4,140 (22.3%)	715 (25.7%)	4,283 (44.6%)	4,998 (40.3%)	1,954 (31.0%)	5,675 (49.9%)	7,629 (43.2%)
**Specialty, N (%)**									
Family Medicine	892 (57.5%)	4,312 (48.2%)	5,204 (49.6%)	1,369 (49.2%)	4,194 (43.7%)	5,563 (44.9%)	3,177 (50.4%)	4,477 (39.4%)	7,654 (43.3%)
All other specialties	658 (42.5%)	4,630 (51.8%)	5,288 (50.4%)	1,412 (50.8%)	5,413 (56.3%)	6,825 (55.1%)	3,125 (49.6%)	6,891 (60.6%)	10,016(56.7%)
**Days worked per year***									
Mean ± SD	265.1 ± 43.4	285.2 ± 45.6	282.2 ± 45.8	239.0 ± 50.4	257.5 ± 49.9	253.3 ± 50.6	234.1 ± 54.0	246.6 ± 54.1	242.1 ± 54.4
Median (IQR)	266 (236-295)	287 (253-321)	284 (250-317)	243 (214-270)	259 (229-291)	255 (226-287)	238 (202-270)	248 (214-283)	245 (209-279)
**Patient visits per year**									
Mean ± SD	6,236.4 ± 3,490.8	7,361.2 ± 4,352.2	7,195.0 ± 4,254.6	5,611.3 ± 3,792.1	6,973.9 ± 4,848.7	6,668.0 ± 4,667.1	4,527.1 ± 3,769.6	5,894.4 ± 5,445.5	5,406.8 ± 4,957.2

© 2025 Kiran T et al. *JAMA Netw Open*.

Median (IQR)	6,060 (3,977-7,819)	6,862 (4,374-9,445)	6,683 (4,311-9,189)	5,303 (2,716-7,488)	6,245 (3,544-9,246)	5,969 (3,351-8,812)	3,717 (2,406-5,451)	4,546 (2,860-7,092)	4,205 (2,686-6,461)
**Patient visits per day**									
Mean ± SD	23.4 ± 12.7	25.5 ± 14.3	25.2 ± 14.1	22.7 ± 13.4	26.3 ± 16.1	25.5 ± 15.6	18.5 ± 12.5	22.8 ± 17.6	21.3 ± 16.1
Median (IQR)	23 (15-29)	24 (16-32)	24 (16-32)	21 (13-30)	24 (15-34)	23 (14-33)	16 (11-22)	18 (13-27)	17 (12-25)
**Total payments from all sources**									
Mean ± SD	234,035 ± 101,817	289,372 ± 127,735	281,197 ± 125,785	306,530 ± 125,892	385,753 ± 172,309	367,968 ± 166,356	428,919 ± 206,235	561,696 ± 311,742	514,341 ± 285,892
Median (IQR)	202,924 (170,770-264,651)	258,539 (201,368-343,889)	249,351 (194,568-334,734)	269,830 (221,278-356,118)	350,508 (266,907-462,790)	331,321 (250,218-440,788)	383,454 (303,408-496,888)	497,858 (374,242-658,987)	451,253 (337,789-602,582)
**Payment per day worked (1 pat min)**									
Mean ± SD	897 ± 405	1,027 ± 458	1,008 ± 453	1,346 ± 763	1,540 ± 771	1,496 ± 773	1,905 ± 1,233	2,324 ± 1,298	2,174 ± 1,291
Median (IQR)	783 (657-1,005)	918 (730-1,195)	896 (716-1,167)	1,159 (937-1,530)	1,368 (1,065-1,800)	1,321 (1,028-1,747)	1,679 (1,342-2,151)	2,054 (1,584-2,704)	1,903 (1,469-2,533)
**Payment per patient visit**									
Mean ± SD	51 ± 46	54 ± 46	53 ± 46	92 ± 176	87 ± 105	88 ± 124	139 ± 159	142 ± 128	141 ± 140
Median (IQR)	33 (28-58)	36 (27-67)	36 (27-66)	56 (36-107)	58 (35-106)	58 (35-106)	104 (78-153)	108 (75-162)	106 (76-158)

* A day worked was defined as any day on which a physician visited a minimum of one patient.

© 2025 Kiran T et al. *JAMA Netw Open*.

eFigure 1. Ratio of Median Total Payments to Male Physicians vs Female Physicians in Full-Time Physicians in 1992-1993, 2005-2006, and 2019-2020, Stratified by Specialty*

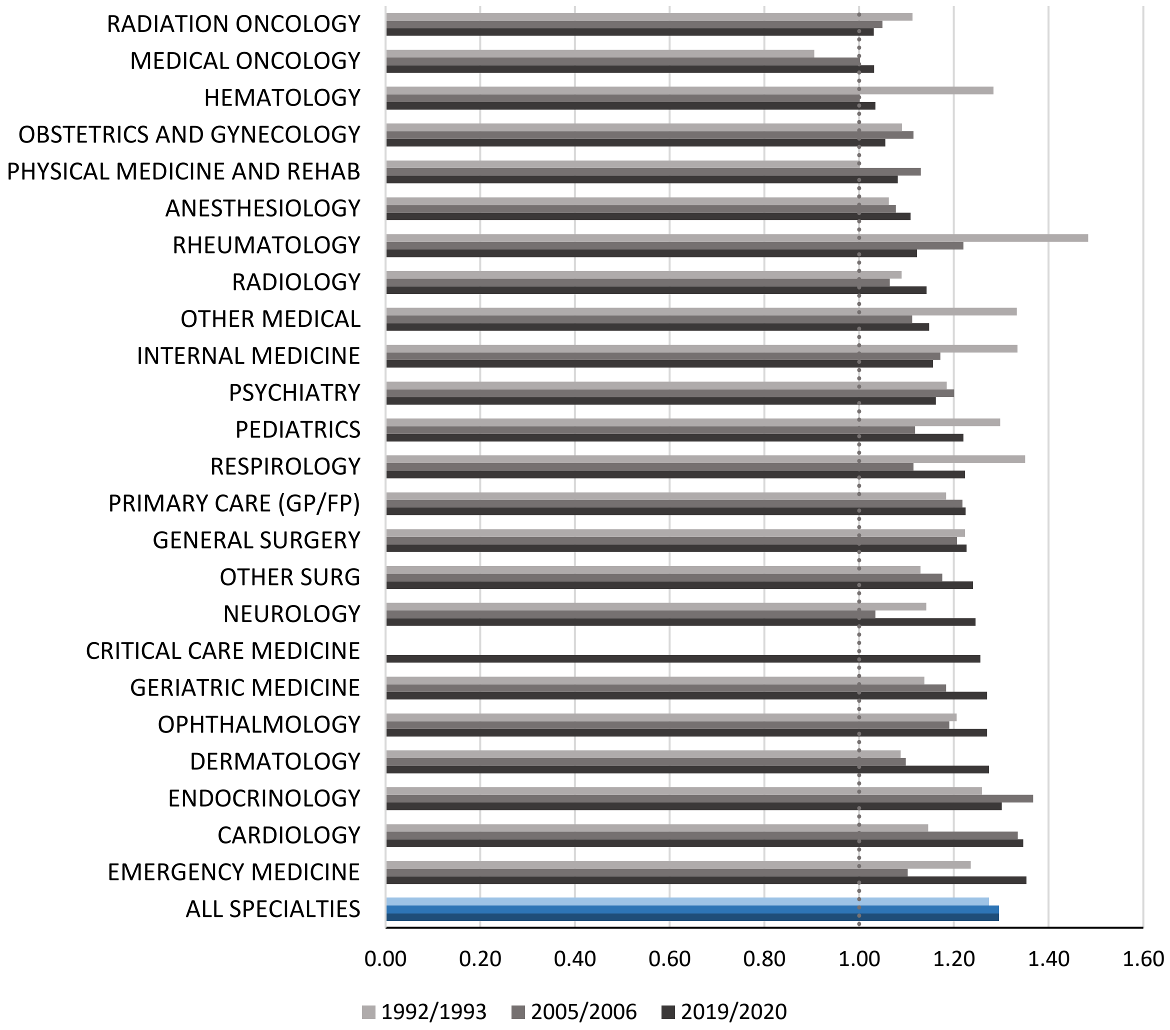

* Only specialties with six and more female physicians are included

© 2025 Kiran T et al. *JAMA Netw Open*.

eFigure 2. Changes in the Percentage of Female Physicians and Median Total Payment in All Physicians in All Years From 1992-1993 to 2019-2020, by Specialty

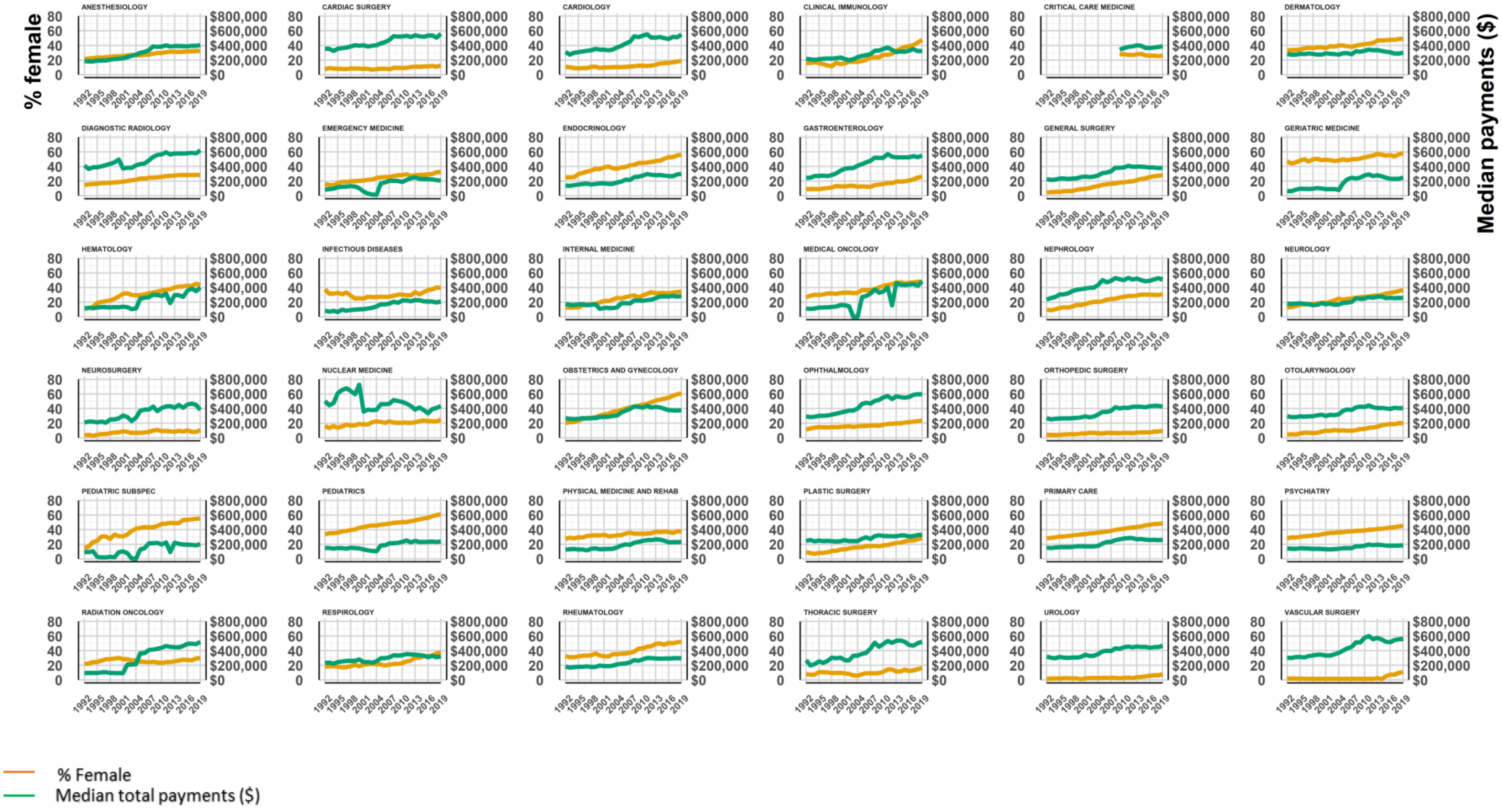

© 2025 Kiran T et al. *JAMA Netw Open*.

eFigure 3. Changes in the Percentage of Female Physicians and Median Total Payment in Full-Time Physicians From 1992-1993 to 2019-2020, by Specialty

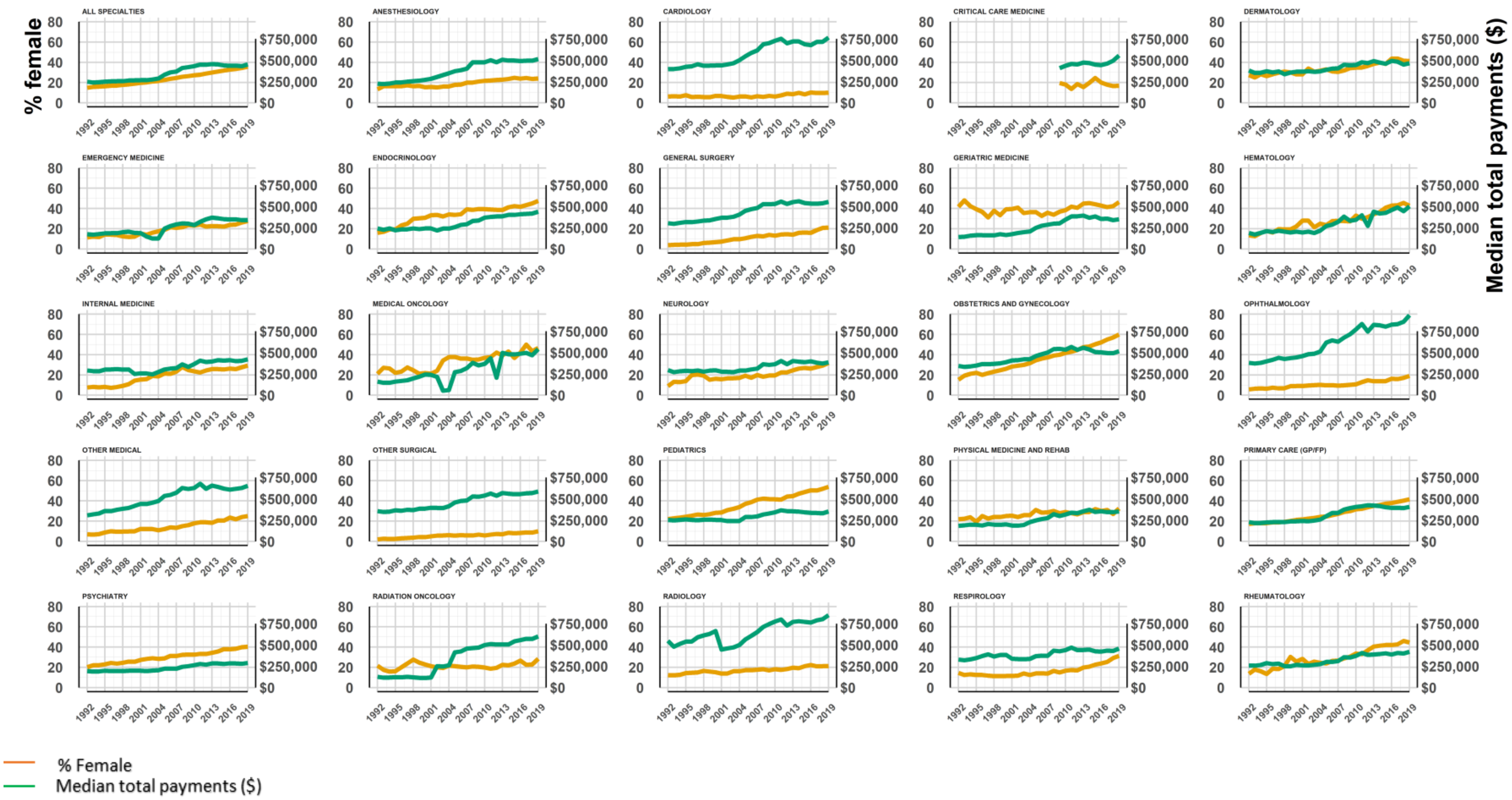

© 2025 Kiran T et al. *JAMA Netw Open*.
